# Functional elbow range of motion 6 months after contracture release and ORIF K-wire in elbow stiffness with malunion capitellum and neglected radial head and ulnar dislocation: a case report

**DOI:** 10.1016/j.ijscr.2019.04.036

**Published:** 2019-05-16

**Authors:** Wahyu Widodo, M. Ade Refdian, Ajiantoro

**Affiliations:** Department of Orthopaedic and Traumatology, Faculty of Medicine Universitas Indonesia, Cipto Mangunkusumo Hospital, Indonesia

**Keywords:** elbow stiffness, contracture release, malunion capitellum fracture, neglected dislocation of radial head and ulnar, functional elbow range of motion

## Abstract

**Introduction:**

Elbow stiffness is the most common complication following trauma of the elbow. This is because the elbow joint is susceptible to effusion, hemarthrosis, scarring, and capsule thickening due to its small intracapsular volume. Surgical treatment is therefore necessary to release soft tissue contracture.

**Case:**

A male teenager was unable to flex his elbow since 1 year prior to admission after falling down during football practice. He didn’t seek any medical treatment and had his elbow massaged since 5 months ago. On physical examination, his right elbow was extended, with flexion-extension range of motion (ROM) of 30^0^ - 0^0^. On the radiograph, there was malunion fracture of left capitellum and neglected posterosuperior dislocation of radial head and ulna. Surgery was performed to release contracture and correct the malunion. Normal activity with functional elbow flexion-extension ROM of 110^0^ - 30^0^ was achieved in 6 months after operation.

**Discussion:**

Elbow stiffness is a challenging case for surgeon, especially in regards of developing good perioperative plan. The aim of treatment for elbow stiffness is to achieve a pain-free and functional elbow ROM.

**Conclusion:**

To achieve functional elbow ROM, surgical treatment was necessary to release the contracture. In addition, the etiology of trauma must be thoroughly investigated and a good rehabilitation program must be integrative to the treatment.

## Highlights

• Elbow joint stiffness is relatively common following injury to the elbow.
• This case is very devastating due to loss of overall arm function due to traumatic elbow stiffness.
• We managed to restore ROM of elbow joint to the functional level after traumatic stiffness.
• This combination technique of surgery provides excellent outcome for the patient.

## 1 Introduction

Elbow motion is essential for upper extremity function and hand movement. Unfortunately, elbow is prone to trauma, and it is susceptible of developing complications such as stiffness and degeneration [1]. Abnormalities of the bone, soft tissue, or combination of both, which may be intra-articular or extra-articular, can cause loss of elbow motion [2,3]. Morrey et al stated that elbow functional range of motion (ROM) for daily activity are 30^0^ - 130° of flexion-extension and 50° of pronation-supination in either direction [1]. Non-surgical treatment is not preferable in restoring elbow’s functional ROM after trauma due to its non-satisfactory result. Surgery is still the treatment of choice because elbow joint release, resection of heterotopic ossification, joint reconstruction or interposition arthroplasty could be performed during operation [4].

The bony framework of the elbow joint are the articulations between the trochlea and capitellum of the humerus with the trochlear notch of the ulna and radial head, respectively. Disruption to this structure by fracture, malunion, nonunion, or dislocation, will reduce elbow ROM [1,5].

Thereby, we present a case of 16-year-old with elbow stiffness due to malunion capitellum and neglected radial head and ulnar dislocation. The patient underwent elbow reconstruction with contracture release, ORIF K-Wire, and ulnar transposition and achieved 110^0^ - 30^0^ of flexion-extension ROM. This case report is reported in line with the most recent criteria for case report: SCARE criteria [6].

## 2 Case

A 16-year-old male complained of inability to flex his left elbow since 1 year prior to admission. One and a half year before, he fell down and hit his elbow during football practice. He felt pain and there was swelling on his elbow. However, he didn't seek for medical treatment. He had his elbow massaged every week for 5 months but there was no improvement. His elbow became fixed in extended position. A month later, he went to an orthopaedic surgeon and underwent x-ray examination which revealed a fracture and dislocation on his left elbow. He was then referred to our institution for further treatment.

**Fig. 1:**
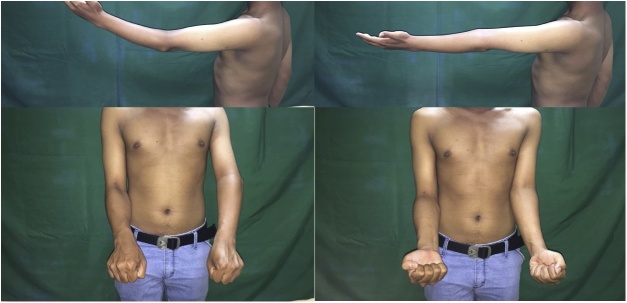
Elbow flexion-extension is limited to 30^0^–0^0^, pronation-supination is normal.

**Fig. 2:**
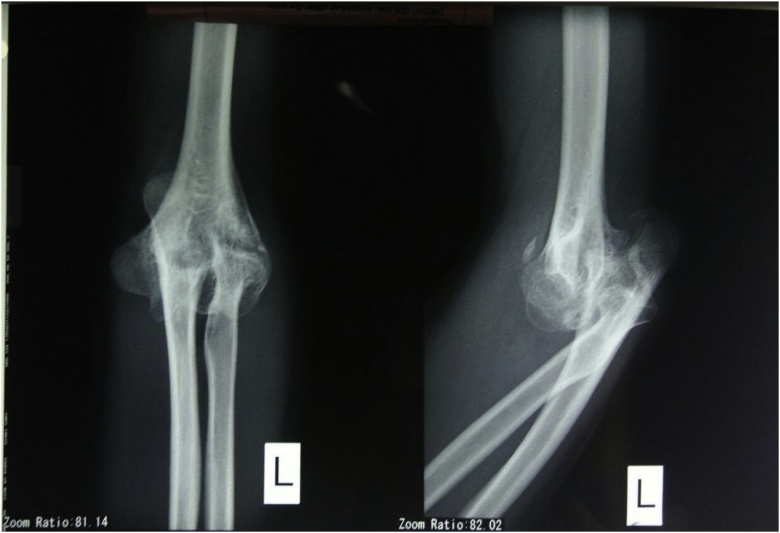
X-ray of the left elbow AP and lateral.

**Fig. 3:**
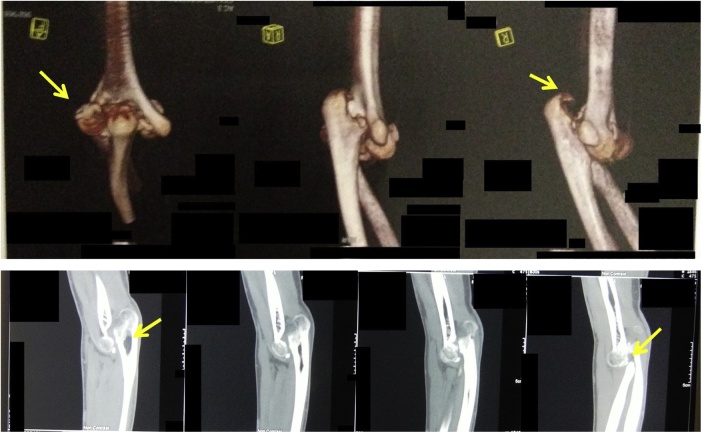
2D and 3D CT Scan reconstruction of the left elbow.

From clinical examination, range of flexion-extension of the elbow was 30^0^-0^0^ with normal pronation-supination. There was no neurological deficit (Fig. 1). From radiological examination, there was a malunion of medial epicondyle with subluxation of left proximal ulna (Fig. 2). From 3D CT reconstruction, there was a deformity and malunion fracture in humeral capitellum with radial and ulnar postero-superior dislocation (Fig. 3). The patient was diagnosed with extension contracture of the left elbow due to malunion of left capitellum, neglected dislocation of the radiohumeral joint, and neglected dislocation of the ulnohumeral joint. The patient was scheduled to have a contracture release, open reduction and internal fixation, and ulnar interposition.

Intraoperatively, we did a posterior approach to the elbow. The ulnar nerve was identified and preserved. The fibrotic tissues and heterotopic ossification were excised. We did a contracture release and open reduction and internal fixation using K-Wire. The flexion and extension of the elbow were evaluated and we managed to get 30^0^ - 130° of flexion-extension ROM. Afterwards, ulnar interposition was performed to prevent ulnar impingement. The wound was closed and a single drain was placed. The elbow was immobilized with back-slab in 90^0^ flexion position for two weeks.

After 1 week, the patient went back to our hospital for follow-up examination. In the 1^st^ evaluation, we tried to remove the back slab and moved the elbow passively. The movement is restricted due to pain and the patient went back home with the back slab on. In the 2^nd^ week follow-up, we permanently remove the back slab and the stitches. At that time, the pain still persisted and the patient was planned to have physical rehabilitation.

On the 4^th^ week after surgery, the surgical wound was infected. We performed debridement, implant removal, and manipulation under general anesthesia. Two weeks later, patient came back to our hospital. We removed the stitches and started rehabilitation. Later on, he continued his rehabilitation in his previous hospital.

**Fig. 4:**
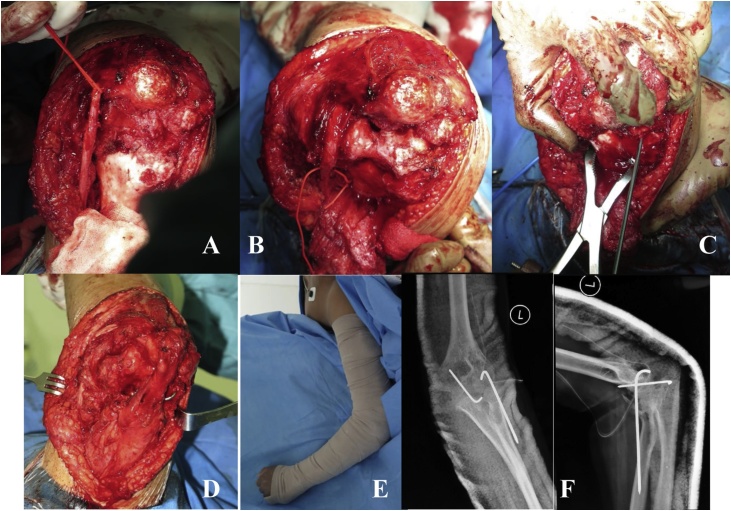
Ulnar nerve and heterotopic ossification identification (A), contracture release and ulnar nerve preservation (B), open reduction and internal fixation using K-Wire (C), final exposed and ulnar nerve transposition (D), immobilization using backslab in 90^0^ flexion position (E), post-operative X-Ray (F).

**Fig. 5:**
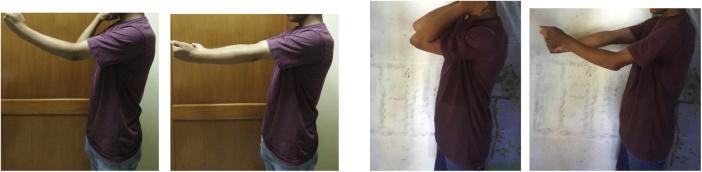
Elbow's ROM comparison preoperative (flexion-extension 30^0^–0^0^) and 6 months postoperative (flexion-extension 110^0^–30^0^).

After 6 months, he visited our outpatient clinic for medical checkup. From physical examination, the elbow flexion-extension ROM was 110^0^ - 30^0^ (Fig. 4). The patient is able to do normal daily activities (Fig. 5).

## 3 Discussion

### 3.1 Elbow's ROM and classification

Elbow stiffness is the most common complication following trauma of the elbow. This is because the elbow is susceptible to effusion, hemarthrosis, scarring, and capsule thickening due to its small intracapsular volume. Normal elbow ROM is up to 0^0^ - 160^0^ in flexion and extension, and 50^0^ in pronation-supination. [1,7] A study by Morrey et al in 1981, showed that people could perform daily activity if the ROM of the elbow was at least 30^0^ - 130^0^ in flexion-extension, and 50^0^ in pronation and supination. Loss of extension greater than 30^0^ and flexion less than 120^0^ is classified as elbow stiffness [3]. Hotchkiss classify elbow stiffness in three categories: minimal, moderate, and severe (Table 1) [8]. Based on this classification, our patient had severe case of elbow stiffness, with ROM of only 30˚.

**Table 1:** Categories of Posttraumatic Stiffness [4].

Category	Range of motion	Likely Outcome
Minimal	Less than 30° of motion loss (usually extension)	Nearly complete recovery of motion
Moderate	40–100° of total motion	Seldom regain full extension
Severe	Less than 30° total motion	30–130 motion achieved Prolonged rehabilitation Strength and power often limited

The etiologies of elbow stiffness are multifactorial. Morrey divided them into three groups based on etiology and anatomical location of the contracture [1,4]. The first group is extrinsic stiffness, of which the elbow ROM is limited due to soft tissues or extra-articular process, e.g. capsular, collateral ligament, and muscle contracture. The second group is intrinsic stiffness, which is related to joint processes adhesion, loose bodies, osteophytes formation, malalignment of the articular surfaces, and degenerative joint disease. The last group is mixed type, in which the intrinsic pathology developed into extrinsic contracture [1,4].

Another classification was described by Kay [2], wherein elbow stiffness is classified into 5 types according to the offending structures: isolated soft tissues contracture (type 1), soft tissues contracture with heterotopic ossification (type 2), nondisplaced articular fracture with soft tissues contracture (type 3), displaced articular fracture with soft tissues contracture (type 4), and post traumatic bony bars (type 5) [4,5].

### 3.2 Treatment

The treatment goal for elbow stiffness is to achieve pain-free, functional ROM, and stable elbow. Non-operative treatment is considered in patients whose onset of elbow stiffness is 6 months or less. Conservative treatment consists of static and dynamic splinting, serial casting, continuous passive movement (CPM), occupational/physical therapy, and manipulation under general anesthesia. In recent studies, botulinum toxin injections can also improve range of motion in children with elbow stiffness [1,4,7].

Patients who have elbow flexion-extension ROM of less than 100^0^ or pronation-supination ROM of less than 50^0^ to –50^0^ are indicated for surgery, either by arthroscopic or open surgery [4]. In severe cases, where medial and lateral exposure are needed, or when the articular joint surface is affected, extensive posterior approach is preferable. Using this approach, we could perform elbow joint release, resection of heterotopic ossification, joint reconstruction or interposition arthroplasty [4].

A study by Sivakumar et al. [9] reported two cases of interpositional arthroplasties (IPA) which were performed in 22-year-old female and 24-year-old male with history of elbow stiffness following a traumatic event. In both cases, by using posterior approach to the elbow, osteolysis and interposition of fascia lata grafts over the recreated articular surfaces were performed. During follow-up, both patients had good range of motion and stability. In comparation with our study, our patient has better range of motion with no limitation in daily activities [9]. IPA is a type of resurfacing surgery which is viable in young patients who have posttraumatic elbow stiffness with intact bony anatomy. In their original study, Cheng and Morrey followed-up 13 patients who underwent IPA. They found that 70% of the patients achieved pain relieve. They concluded that IPA is a useful option for young high demand patients with arthritis of the elbow. Nevertheless, it may not be useful in generalized inflammatory arthritis. This procedure has its own drawbacks, including elbow instability, fascia rupture, thigh pain, and neuropraxia [9].

The problems of our patient is the one-year onset of extension contracture, which is accompanied by malunion fracture of capitellum an neglected radial head and ulna dislocation. We performed soft tissue release, open reduction and internal fixation, heterotopic ossification removal, and ulnar transposition.

Fractures of the capitellum is rare in adolescents over the age of 12 years, therefore it is often missed on daily practice [10,11]. Plain radiograph often underestimates the degree of fracture complexity. Thus, reconstructive imaging technique like CT scan is more preferable. Bryan and Morrey [12] classified this fracture into three types: Hahn-Steinthal fracture (type 1) which involves large part of the bony portion and subchondral bone of capitellar; Kocher-Lorenz fracture (type 2) which involves articular cartilage with very little subchondral bone attached; and Broberg and Morrey fracture (type 3) in which the capitellar is comminuted. Later, McKee modified this classification system and add the 4^th^ type, which is known as coronal shear fracture [10,11]. By using the modified classification, our case is classified into type 1 fracture.

Various internal fixation methods have been introduced for treatment of capitellum fracture, including K-Wires, 4 mm cancellous screws, Headless screw, absorbable polyglycide pins, and plate fixation [10,13,14]. In our case, we performed open reduction and internal fixation using K-Wire. The K-Wire itself did not provide enough stability for mobilization until fracture healing. Therefore, we used back slab in flexion position, and started rehabilitation after 2 weeks when soft callus had appeared [10]. Compared to the screw, the K-wire provides better functional outcome. The duration of this procedure is also shorter [13]. In addition, screw was not preferred because the erosion of radial head with screw can lead to avascular necrosis or chondrolysis [10].

We performed open reduction for neglected elbow dislocation. Lyons et al. [5] stated that the standard treatment for chronic elbow dislocation consisted of open reduction, v-y-muscleplasty of the triceps, and immobilization using cast. Devnani showed that open reduction of chronic elbow dislocation with capsule and fibrous adhesions excision, followed by ulnar nerve transposition without collateral ligament reconstruction, improves elbow ROM and function, as well as elbow stability, regardless of collateral ligaments excision [15]. The ulnar nerve transposition was performed to prevent ulnar nerve compression by excessive fibrous tissue [1]. The nerve was transposed to anterior position and protected by fascia flap. We made sure there was no tension, compression, or any kinks. The joint was flexed and extended to make sure the nerve is in good position [8]. In case where the patient clinically presents with ulnar nerve neuropathy syndromes, neurolysis and nerve transposition should be performed [4].

We also performed removal of heterotopic ossification. This condition is characterized by formation of mature lamellar bone in nonosseous tissue. It restricts elbow motion and upper extremity function [2]. Heterotopic ossification has been considered as poor prognostic factor in elbow joint stiffness [4].

Finally, after all the procedure, we evaluated the elbow ROM and achieved full flexion-extension of 140-0^0^. We immobilized the elbow in 90^0^ flexion position using back slab. In other study, splinting is used to add stability to K-Wire fixation [10]. It is also recommended to haul soft tissues to increase ROM, in the absence of heterotopic ossification. Study by Gallucci et al reported that over 60% of patients using splint attain functional ROM [7].

Regaining elbow ROM, restoring muscle strength, and reintegrating the arm into daily life are the aims of postoperative management and rehabilitation programs, and should be carried out until no further enhancement are made [2]. Most surgeons start early mobilization within 48 hours after open capsular release. However, in previous European study, more than 60% cases of elbow dislocation were treated with cast immobilization for 3 weeks to prevent instability due to early movement [7]. A Study by Lordens et al. [16] reported that early mobilization resulted in earlier functional recovery compared to immobilization group. However, there is no significant difference in functional ROM, statistically [17].

## 4 Conclusion

ORIF and K-wire with contracture release is a viable treatment option for traumatic elbow stiffness due to malunion of capitellum and neglected radial and ulnar head dislocation. This procedure can help in achieving functional ROM.

## Conflicts of interest

Nothing to declare.

## Funding

Nothing to declare

## Ethical approval

This is a case report; appropriate informed consent has been obtained from the parent of the patient. Ethical approval for this case report has been exempted by our ethical committee.

## Consent

Appropriate informed consent has been obtained for publication of this case report along with the accompanying images. A copy of written consent is available for review on request.

## Author contribution

M Ade Refdian, Wahyu Widodo – examining patient, following up patient, writing the manuscript up and reviewing literatures.

Ajiantoro – editor of the content of the manuscript and language editor.

Wahyu Widodo – reviewing and giving approval of the manuscript, also senior orthopaedic surgeon assigned to this case.

## Registration of research studies

Not available.

## Guarantor

Wahyu Widodo, M.D. (corresponding author)

Orthopaedic Surgeon, Hand Consultant at Department of Orthopaedic and Traumatology, Faculty of Medicine Universitas Indonesia.

## Provenance and peer review

Not commissioned, externally peer-reviewed
